# SWCTI: Surface Water Content Temperature Index for Assessment of Surface Soil Moisture Status

**DOI:** 10.3390/s18092875

**Published:** 2018-08-31

**Authors:** Zhiming Hong, Wen Zhang, Changhui Yu, Dongying Zhang, Linyi Li, Lingkui Meng

**Affiliations:** 1 School of Remote Sensing and Information Engineering, Wuhan University, Wuhan 430079, China; simonhong@whu.edu.cn (Z.H.); wen_zhang@whu.edu.cn (W.Z.); ych@whu.edu.cn (C.Y.); lilinyi@whu.edu.cn (L.L.); 2 School of Water Conservancy and Environment, Zhengzhou University, Zhengzhou 450001, China; zhangdongying@whu.edu.cn

**Keywords:** Moderate Resolution Imaging Spectroradiometer (MODIS), SWCTI, SWCI, VSWI

## Abstract

The vegetation supply water index (VSWI = NDVI/LST) is an effective metric estimating soil moisture in areas with moderate to dense vegetation cover. However, the normalized difference vegetation index (NDVI) exhibits a long water stress lag and the land surface temperature (LST), sensitive to water stress, does not contribute considerably to surface soil moisture monitoring due to the constraints of the mathematical characteristics of VSWI: LST influences VSWI less when LST value is sufficiently high. This paper mathematically analyzes the characteristics of VSWI and proposes a new operational dryness index (surface water content temperature index, SWCTI) for the assessment of surface soil moisture status. SWCTI uses the surface water content index (SWCI), which provides a more accurate estimation of surface soil moisture than that of NDVI, as the numerator and the modified surface temperature, which has a greater influence on SWCTI than that of LST, as the denominator. The validation work includes comparison of SWCTI with in situ soil moisture and other remote sensing indices. The results show SWCTI demonstrates the highest correlation with in situ soil moisture; the highest correlation R = 0.801 is found between SWCTI and the 0–5 cm soil moisture in a sandy loam. SWCTI is a functional and effective method that has a great potential in surface soil moisture monitoring.

## 1. Introduction

Soil moisture plays an important role in the conversion of water and energy among the hydrosphere, biosphere, and atmosphere. Soil moisture is a key input parameter for hydrological models, climate models, ecological models, and land surface process models [1] and is widely used in other many studies and applications [2]. It is of great importance to improve the accuracy of soil moisture estimation. Over the last few decades, numerous methods, including field measurement methods and remote sensing methods—based on visible (VIR), near infrared (NIR), shortwave infrared (SWIR) reflectances, thermal infrared (TIR) emittance, and microwave emissions—have been developed to estimate soil moisture. The field measurement methods, including time domain reflectometry (TDR), dielectric constant, and capacitance probe methods, are the most traditional methods. These methods provide highly precise point-based measurements but are difficult to use on a large scale because of the high spatial variability of soil moisture, sparsity of soil moisture stations, and expense. Fortunately, remote sensing techniques provide an approach to monitor soil moisture at higher temporal and spatial resolutions with lower cost and time requirements [3]. Among the remote sensing methods, microwave-based methods are the most effective for estimating soil moisture [4]. Microwave technology has many advantages, for example, it can operate day under all weather conditions, even penetrating through clouds and vegetation. While passive microwave methods have the disadvantage of low spatial resolution (20~50 km), active microwave methods can provide monitoring products at higher spatial resolutions, but the temporal resolution of active microwave methods is relatively low (16~25 days). Generally, the VIR, NIR, MIR, and TIR data provided by MODIS are greatly influenced by the atmosphere and clouds but have relatively high temporal and spatial resolution; additionally, the acquisition of this data is simple and inexpensive. Therefore, MODIS data can be used as an important means for supplementing microwave soil moisture monitoring methods.

In the study of surface soil moisture estimation, many remote sensing methods have been developed based on VIR, NIR, SWIR, and TIR data. Ghulam [5] proposed the perpendicular drought index (PDI) on the basis of the spatial characteristics of the soil moisture distribution in NIR–red space, which is applicable to areas covered by only bare soil or low vegetation. On the basis of PDI, Ghulam [6] introduced the fractional vegetation cover (FVC) and developed the modified perpendicular drought index (MPDI). Compared with PDI, MPDI is more suitable for monitoring soil moisture in areas with high vegetation cover. Similarly, Yao [7] used the MODIS SWIR Band 6 and Band 7 reflectances to develop the shortwave infrared soil moisture index (SIMI), and Dong [8] proposed the shortwave infrared water stress index (MSIWSI) using the spectral feature space obtained from the MODIS Band 6 and Band 7 reflectances, the SWIR reflectances, and FVC. Fensholt [9] proposed the shortwave infrared water stress index (SIWSI) using NIR and SWIR by considering that leaf water is highly absorptive in SWIR wavelengths. Du [10] proposed the surface water content index (SWCI) by normalizing the difference of MODIS Band 6 and Band 7 data. Zhang [11] developed the shortwave infrared drought index (VSDI) using SWIR, green, and red channels. Wang [12] proposed the normalized multiband index (NMDI). Both land surface temperature (LST) and the normalized difference vegetation index (NDVI) have complicated dependencies on soil moisture. When the vegetation water supply is normal, NDVI and LST are stable and vary only slightly within a certain range. When the vegetation water supply is insufficient, NDVI decreases, while LST increases. Previous studies have found that NDVI-LST feature space manifests as a triangular shape [13,14] or a trapezoidal shape [15] when changes in vegetation cover and soil moisture conditions in the study area are large enough. Sandholt [16] proposed the temperature–vegetation dryness index (TVDI) by combining NDVI with LST based on observations from the LST-NDVI feature space. However, TVDI is applicable only if most of the study area is cloudless and if this cloudless area is sufficiently large (from wet bare soil to dry bare soil, from water-stressed vegetation to well-watered vegetation) [3]. However, these conditions are rare, and the choice of regional scope has a great impact on TVDI. The vegetation supply water index (VSWI) is also a relatively simple and effective metric to estimate surface soil moisture and has been shown to be significantly related to crop moisture content and soil moisture content under most climate types and land cover types [17]. However, NDVI is easily affected by soil lines in areas with low vegetation cover, and it tends to saturate in areas with high vegetation cover and exhibits a long water stress lag [18]. These characteristics of NDVI will reduce the sensitivity of VSWI to the changes in soil moisture. In contrast, surface temperature is relatively sensitive to water stress [19]. However, due to the mathematical characteristics of the VSWI ratio, LST contributes less to the sensitivity of VSWI. 

This paper aims to provide an operational and effective method for estimating surface soil moisture in areas with high vegetation cover by proposing the surface water content temperature index (SWCTI), which is developed by combining the water-sensitive SWCI with the modified surface temperature. To validate SWCTI, we analyze the relationship between SWCTI and in situ soil moisture and compare SWCTI with other commonly used remote sensing soil moisture indices, including VSWI, TVDI, SIWSI, NMDI, and SWCI.

## 2. Materials and Methods

### 2.1. Study Area

Naqu City lies in the central Tibetan Plateau (83°55′ E~95°5′ E, 25°55′ N~36°30′ N) among the Tanggula Mountains, Nyainqentanglha Mountains, and Gangdese Mountains and has a total area of 369,674 km². Naqu City is in a dry and windy subfrigid climate zone. The annual average temperature is −0.9 °C to −3.3 °C, the annual relative humidity ranges from 48% to 51%, the annual precipitation is 380 mm, and the annual sunshine hours range from 2852.6 to 2881.7 h. There is no completely frost-free period throughout the year. From November to March of the following year, the climate is dry, and the temperature is low. From May to September, the climate is mild, the weather is calm, and the rainfall during this period accounts for 80% of the rainfall during the whole year. The area with the highest precipitation is located in southeastern Naqu, where the annual precipitation exceeds 580 mm, while the area with the lowest precipitation is in northwestern Naqu City, where the annual precipitation is less than 440 mm. In this study area, the multiscale Soil Moisture and Temperature Monitoring Network on the central Tibetan Plateau (CTP-SMTMN) was established, consisting of 57 stations, and can provide field-measured soil moisture data for our validation work [20,21].

### 2.2. Data

#### 2.2.1. In Situ Soil Moisture Measurements

**Figure 1:**
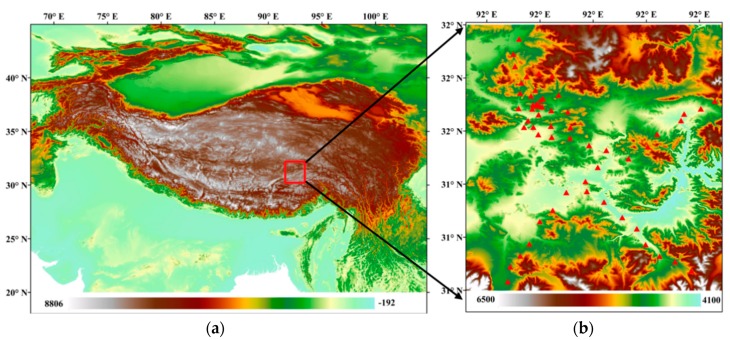
Spatial distribution of the 57 stations in Naqu City, the Tibetan Plateau. (**a**) The location of CPT-SMTMN in the Tibet Plateau; (**b**) The detailed site distribution within CPT-SMTMN. Red triangles present the stations.

CTP-SMTMN soil moisture data provided by the Institute of Tibetan Plateau Research (ITP), Chinese Academy of Sciences (CAS), are used as the validation data. CTP-SMTMN is located in ~100 × 100 km of Naqu City (31°~32° N, 91.5°~92.5°) in the central Tibetan Plateau, with a mean elevation of 4650 m. This area has rolling hills but is generally relatively flat, although the topography is rugged in some places. CTP-SMTMN includes 57 observation sites (see Figure 1 and Table A1 in Appendix A), which provide soil moisture data at four depths (0~5, 10, 20, and 40 cm). The vegetation in this area is mainly alpine meadow, and the organic matter in the soil under the meadow is high [20]. Here, soil carbon is mainly found in the topsoil (the average content is 3.6%) and gradually decreases with the increase in soil depth (approximately 0.6% at 40 cm). Silty and sandy soil are the main soil components (50% and 46%, respectively), and the silt content remains below 10%. The field measured data selected for our validation work are consistent with the MODIS transit time [21].

#### 2.2.2. Terra MODIS Data

We used a Terra MODIS (Version 5) eight-day land surface reflectance (LSR) product (MOD09A1) and an LST product (MOD11A2) from 1 July to 30 September, 2010–2014 (except July, 2010 when in situ soil moisture data is not available), to calculate remote sensing indices. This open access data can be downloaded from https://lpdaac.usgs.gov. The MOD09A1 product provides an estimate of the surface spectral reflectance of Terra MODIS Bands 1–7. A quality layer is included with the seven 500 m solar reflectance bands. The MOD11A2 product provides an eight-day average per-pixel LST with a spatial resolution of 1 km. 

To guarantee MODIS data quality, data quality control is required. For each pixel of the 1–7 bands of MOD09A1, quality control was carried out by using the Land Data Operational Products Evaluation (LDOPE) mask_sds tool to eliminate the pixels affected by clouds and high aerosols. The quality control data were from the surf_refl_state_500 m quality layer in MOD09A1. The quality control standards are listed in Table 1. For a given pixel, if the bit combination located in bit number 0–1, 2, 6–7, 8–9, 12, and 13 are 00, 0, 01, 00, 0 and 0, respectively, then the pixel will be kept, otherwise, the pixel will be excluded. Similarly, for each pixel of MOD11A2 LST data, this work used the mask_sds tool of LDOPE to carry out the quality control operation and selected the pixels with good quality. The quality control data were from the MOD11A2 LST_Day_1 km data, and the quality control standards are shown in Table 1. For a given pixel, if the bit combination located in bit number 0–1 is 00 or 01, then the pixel will be kept, otherwise, the pixel will be excluded. Meanwhile, because the spatial resolution of the reflectance products of Bands 1–7 in MOD09A1 and LST in MOD11A2 were not consistent, the reflectance layer in MOD09A1 was resampled to 1 km using the cubic convolution method.

#### 2.2.3. In Situ Meteorological Data

The daily precipitation data from 2010 to 2014 from the available stations corresponding to the MODIS data acquisition time were acquired from ITP, CAS (http://www.tpedatabase.cn). However, only one meteorological station is located in the study area of Naqu City. In addition, the precipitation data were used to validate the sensitivity of the proposed index to the rainfall events.

### 2.3. Technical Approach

#### 2.3.1. SWCTI

**Figure 2:**
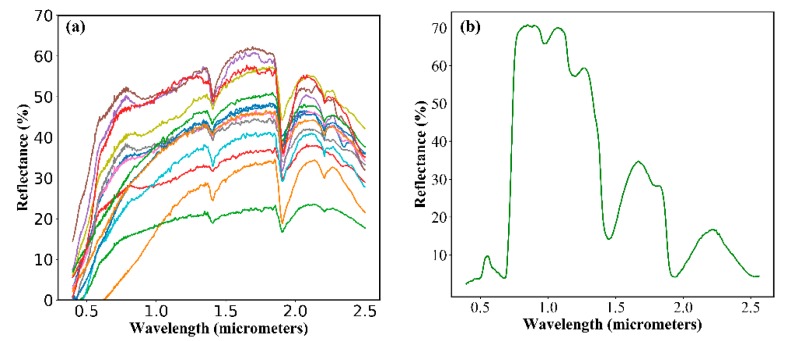
(**a**) Spectral response curves of 14 kinds of soil, provided by the ASTER Spectral Library [22]; (**b**) spectral response curve of green vegetation.

Soil is a mixture of multiple elements, the spectral properties of which are a comprehensive reflection of its components. The spectral properties of soil are influenced by many factors such as soil color, organic matter content, and soil moisture. For a single type of soil, the spectrum is greatly limited by soil moisture [10]. It is known that water exhibits a variable absorption of light in the 1.1~2.4 μm wavelength range. In this spectrum, there are two strong absorption peaks near 1.45 μm and 1.9 μm and an absorption valley near 1.65 μm. Although the spectral properties of different soils are slightly different, in general, the soil reflectance will decrease with the increase in soil moisture, and two absorption peaks form near the wavelengths of 1.45 μm and 1.9 μm. Figure 2a shows the spectral reflectance curves of 14 kinds of soil, provided by the ASTER Spectral Library, including very dark grayish silty loam, grayish brown loam, dark grayish brown silty loam, pale brown silty loam, dark brown fine sandy loam, gray silty clay, reddish brown fine sandy loam, brown sandy loam, brown to dark brown gravelly loam, brown to dark brown gravelly fine sandy loam, brown silty loam, brown loamy fine sand, brown fine sandy loam, and gray/dark brown extremely stoney coarse sand [22]. The reflectance curves of the soils show that the reflectance first increases with the wavelength; then, reflectance valleys are observed near the wavelengths of 1.45 and 1.9 μm. In addition, the reflectance peak is observed near the wavelength of 1.65 μm. The vegetation spectrum in the wavelength of 1.4~2.4 μm is basically controlled by water. The reflectance of vegetation decreases with the increase in leaf water content. Additionally, two reflectance valleys are also observed near 1.45 μm and 1.9 μm; these locations match the absorption valleys of water. There is also an absorption peak near 1.65 μm (see Figure 2b) in the green vegetation spectrum. According to the abovementioned spectral characteristics, water is an important factor affecting the spectral characteristics of both soil and vegetation. There is a peak in soil and vegetation near 1.65 μm, while there are absorption valleys at 1.45 and 1.95 μm. Therefore, the difference in the reflectance at the wavelengths of 1.65 μm and 1.95 μm can be used as an important indicator for water content. 

The MOD09A1 Band 6 and Band 7 reflectances approximately correspond to the absorption valleys and peaks of water; the reflectance of green vegetation and soil at Band 6 is greater than that at Band 7, and the difference between these two bands can be used to monitor water content in vegetation and soil. Because both Band 6 and Band 7 have the same atmospheric scattering and radiation values, the influence of the atmospheric noise can be reduced by normalizing the difference in the bands. Based on this information, SWCI was proposed by Zhang [10] to monitor the surface water content.

(1) $$\;\mathrm{SWCI}=\frac{B_{6}-B_{7}\;}{B_{6}+B_{7}}$$

where $B_{6}$ and $B_{7}$ represent the reflectances of MOD09A1 Band 6 and Band 7, respectively.

When the surface soil moisture increases, vegetation roots absorb water from the soil, leading to an increase in the vegetation water content. Consequently, the difference between the two bands increases, causing SWCI to increase. Therefore, SWCI is positively related to soil moisture. Because these two bands are located in the water absorption area and are sensitive to the change in water [9], it is possible for SWCI to monitor soil moisture sensitively. Compared with NDVI, SWCI directly reflects the water content in vegetation, while NDVI reflects the water content in vegetation on the basis that the characteristics of chlorophyll strongly absorb red light and reflect NIR. SWCI is a more direct indicator, which is beneficial to improve the estimation accuracy of soil moisture. Many studies show that SWCI has a higher correlation with surface soil moisture than that of NDVI [11,23]. 

LST is an important indicator of soil moisture status, which is sensitive to water stress. The vegetation canopy absorbs solar radiation and converts it into heat energy, which leads to increasing canopy temperature. The vegetation consumes this heat through transpiration, lowering the canopy temperature. When the soil water is insufficient, the decrease in water supply leads to a decrease in vegetation transpiration and heat energy consumed, increasing canopy temperature. When the soil water is sufficient, the canopy temperature is relatively low; therefore, the soil moisture under the same vegetation cover condition can be monitored using LST. 

The combination of NDVI and LST (VSWI) is more sensitive to soil moisture than the single indices NDVI and LST. Similarly, the combination of SWCI and LST is expected to be more sensitive to soil moisture than SWCI and LST and is more effective than VSWI because of the advantage of SWCI over NDVI.

Let us assume the following combination

(2) $$\;F\left(\mathrm{SWCI},\mathrm{LST}\;\right)=\frac{\mathrm{SWCI}}{\mathrm{LST}}$$

where F is a function of the variables SWCI and LST, similar to VSWI. 

**Figure 3:**
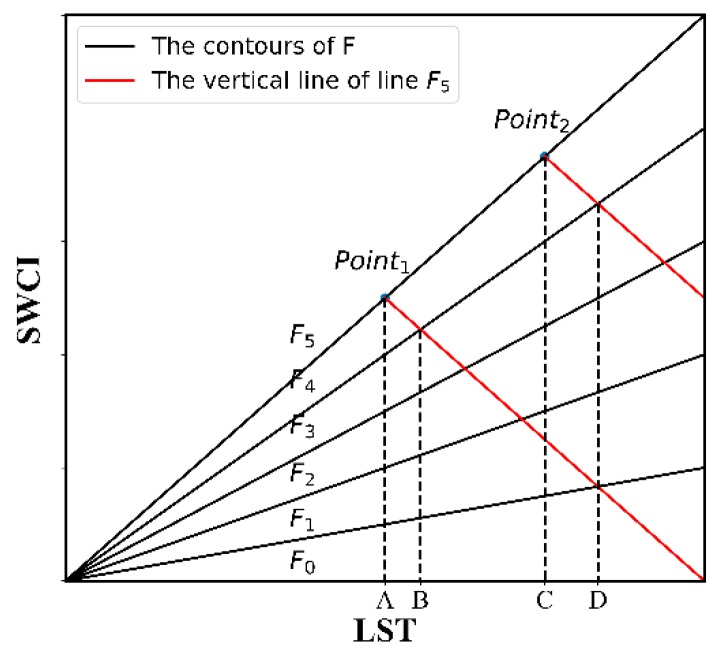
Contours of F.

Figure 3 shows the contour of F, where $F_{i}$ is one of the isolines. Taking $F_{5}$ as an example, the red line is the vertical line of $F_{5}$. Figure 3 shows that for $\Delta F\;={\;F}_{4}-F_{5}$, ΔF at ${\mathrm{Point}}_{1}$ is lesser than that at ${\mathrm{Point}}_{2}$, meaning that the effect of changes in LST on F decreases as LST increases. 

Mathematically, another expression of F is

(3) $$\;F\left(\mathrm{SWCI},T\;\right)=\frac{\mathrm{SWCI}}{C+T}$$

(4) LST = C + T

where C is a reference or an adjustment factor, the range of which could be obtained from the multi-year statistics of temperature in Naqu City. T is the LST variable.

The partial derivatives of F with respect to T and SWCI are

(5) $$\;\frac{\partial F\;}{\partial T}=-\frac{\mathrm{SWCI}}{C+T^{2}}$$

(6) $$\;\frac{\partial F\;}{\partial \mathrm{SWCI}}=\frac{1}{C+T}$$

Then,

(7) $$\;\Delta F=\frac{\partial F\;}{\partial T}\cdot \Delta T+\frac{\partial F}{\partial \mathrm{SWCI}}\cdot \Delta \mathrm{SWCI}$$

(8) $$\;\Delta F_{T}=\frac{\partial F\;}{\partial T}\cdot \Delta T$$

(9) $$\;\Delta F_{\mathrm{SWCI}\;}=\frac{\partial F}{\partial \mathrm{SWCI}}\cdot \Delta \mathrm{SWCI}$$

here, ΔT is the change in variable T, and ΔSWCI is the change in variable SWCI. $\Delta F_{T}$ is the change in ΔF contributed by ΔT, and $\Delta F_{\mathrm{SWCI}}$ is the change in ΔF contributed by ΔSWCI. 

(10) $$\;\frac{\Delta F_{T}\;}{F}=-\frac{\Delta T}{C+T}$$

(11) $$\;\frac{\Delta F_{\mathrm{SWCI}\;}}{F}=-\frac{\Delta \mathrm{SWCI}}{\mathrm{SWCI}}$$

As shown in Equation (10), the lesser C is, the greater $\frac{\Delta F_{T}}{F}$, and the change in temperature has a greater effect on F; consequently, F is more sensitive to the soil moisture contributed by LST. Moreover, the more sensitive of SWCI to the change in soil moisture, the greater $\frac{\Delta F_{\mathrm{SWCI}}}{F}$, similarly, F is more sensitive to the soil moisure contributed by SWCI. 

Therefore, a new remote sensing index, SWCTI, was proposed. The wetter the soil is, the higher SWCI and the lower LST-C for a certain C, increasing SWCTI.

(12) $$\;\mathrm{SWCTI}=\frac{\mathrm{SWCI}\;}{\mathrm{LST}-C}$$

#### 2.3.2. VSWI

VSWI is a dryness index equal to the ratio of NDVI to LST and can be written as

(13) $$\;\mathrm{VSWI}=\frac{\mathrm{NDVI}\;}{\mathrm{LST}}$$

When the vegetation water supply is normal, NDVI and LST remain within a certain range for a certain period of time. If there is not sufficient water supply to vegetation, the vegetation growth status will be affected, and NDVI will decrease. Additionally, if the vegetation does not supply enough water for evaporation, the leaves will close some of their stomata, causing the temperature of the vegetation canopy to rise. Therefore, when soil moisture decreases, VSWI decreases, and when soil moisture increases, VSWI increases. Because the temperature of the vegetation canopy is difficult to obtain, it is generally replaced by LST.

#### 2.3.3. TVDI

TVDI is another dryness index developed based on the NDVI-LST feature space for estimating surface soil moisture status. If a region (from dry to wet soil, from bare soil to full vegetation cover) is sufficiently large, the NDVI-LST distribution is triangular. Sandholt [16] proposed TVDI based on this feature.

(14) $$\;\mathrm{TVDI}=\frac{T_{s}-T_{\min\;}}{T_{\max}-T_{\min}}$$

where $T_{s}$ represents the surface temperature, $T_{\min}$ represents the wet edge temperature, and $T_{\min}$ represents the dry edge temperature in the NDVI-LST triangle space. TVDI increases as $T_{s}$ approaches the dry edge, meaning that the drought is more severe and the surface soil moisture is lower; in contrast, as $T_{s}$ approaches the wet edge, TVDI decreases, meaning that the soil is wetter.

#### 2.3.4. SIWSI

SIWSI is an index for vegetation moisture monitoring and is determined by combining the NIR reflectance information with the shortwave reflectance information proposed by Fensholt [9]

(15) $$\;\mathrm{SIWSI}=\frac{R_{6}-R_{2}\;}{R_{6}+R_{2}}$$

where $R_{2}$ and $R_{6}$ represent the reflectances of MOD09A1 Band 2 and Band 6, respectively. An SIWSI value above zero means that the reflectance from Band 6 is higher than that from Band 2, indicating canopy water stress. A low SIWSI value is a consequence of sufficient water in the canopy for photosynthetic activity. The higher SIWSI is, the more severe the water stress on the vegetation. 

#### 2.3.5. NMDI

The NMDI is a normalized multi-band drought index for monitoring soil and vegetation moisture. It uses the difference between two liquid water absorption bands (1.64 μm and 2.13 um) as the soil and vegetation water-sensitive band. Strong differences between two water absorption bands in response to soil and leaf water content give this combination the potential to estimate the water content of both soil and vegetation [12].

(16) $$\;\mathrm{NMDI}=\frac{R_{2}-\left(R_{6}-R_{7}\;\right)}{R_{2}+\left(R_{6}-R_{7}\right)}$$

where $R_{2}$, $R_{6}$, and $R_{7}$ represent the reflectances of MOD09A1 Band 2, Band 6, and Band 7, respectively. 

## 3. Results and Discussion

### 3.1. Analysis of the Effect and Optimization of the Variable C

To analyze the effect of C on the proposed index-SWCTI, this work used the in situ data at soil depths of 0–5 cm and 0–10 cm collected in August and September in 2010 and in July, August, and September from 2011 to 2014 to calculate the evaluating index $\Delta R^{2}\;$(see Equation (17)). $\Delta R^{2}$ is used to analyze the effect of C on SWCTI and for the optimization of C. As shown in Equation (17), the higher $\Delta R^{2}$ is, the higher the correlation between SWCTI and the in situ data compared to F (F = SWCTI(C = 0)), in other words, when the correlation between SWCTI and the in situ soil moisture increases, $R_{C}^{2}-R_{C=0}^{2}$ increases and $R_{C=0}^{2}$ remains constant, hence the $\Delta R^{2}$ increases, therefore, the optimal C value can be determined when the $\Delta R^{2}$ reaches its maximum. 

**Figure 4:**
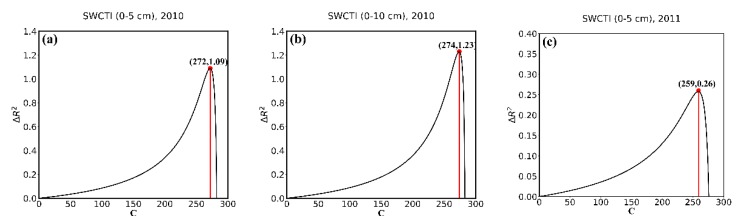

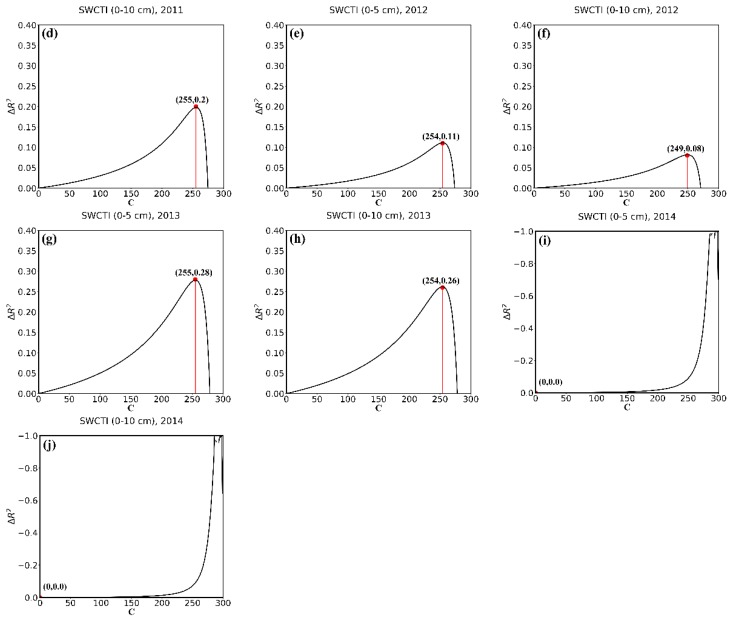
Curves of $\Delta R^{2}$ calculated by using the in situ soil moisture data at different soil depths collected in July, August, and September, (**a**) 0–5 cm in 2010, (**b**) 0–10 cm in 2010, (**c**) 0–5 cm in 2011, (**d**) 0–10 cm in 2011, (**e**) 0–5 cm in 2012, (**f**) 0–10 cm in 2012, (**g**) 0–5 cm in 2013, (**h**) 0–10 cm in 2013, (**i**) 0–5 cm in 2014; and (**j**) 0–10 cm in 2014.

**Figure 5:**
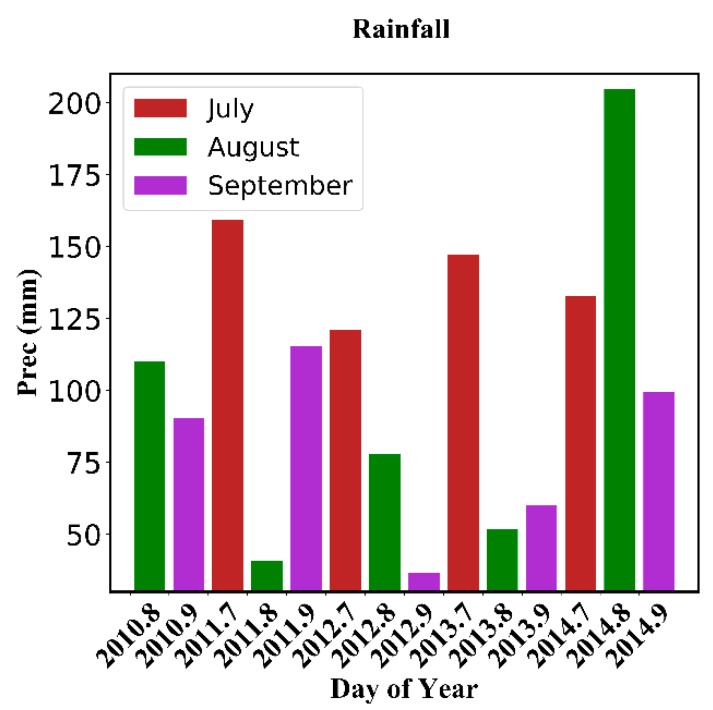
Rainfall in July, August, and September from 2010–2014.

Figure 4 shows the curves of $\Delta R^{2}$ calculated by using the in situ soil moisture data and MODIS data in July, August, and September from 2010 to 2014. As shown in these graphs, $\Delta R^{2}$ first increases and then decreases with the increase in C, reaching a maximum between 249 and 274 for each year except 2014. The $\Delta R^{2}\;$results range from 8% to 123%, proving that the choice of C has a great effect on the performance of SWCTI; therefore, an appropriate value of C is required to improve the proposed index. For 2014, the rainfall in 2014 was significantly high (see Figure 5), and the correlation between LST and soil moisture was low with a coefficient of determination $R^{2}<0.001,\;$which may be responsible for the abnormal changes in the curves in 2014 (see Figure 4i,j).

(17) $$\;\Delta R^{2}=\frac{R_{C}^{2}-R_{C=0\;}^{2}}{R_{C=0}^{2}}$$

here, $R_{C}^{2}$ is the coefficient of determination $R^{2}$ of SWCTI, and $R_{C=0}^{2}$ is the case when C = 0.

**Figure 6:**
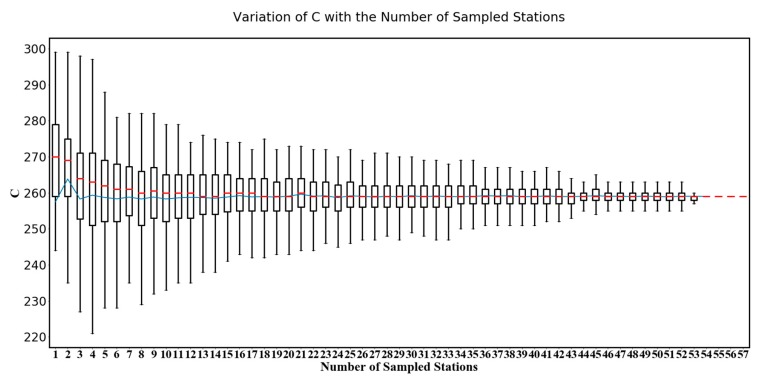
Boxplot which represents variation of C with the number of sampled stations, the red line represents the median of data within the box body and the blue line represents the mean of data within the box body.

Equation (17) shows that the optimization of C is related to the distribution of LST. Therefore, variables which has an impact on LST also have an indirect impact on the optimization of C. These variables may include meteorological factors such as cloud, rainfall, etc. Moreover, the determination of the optimal C is related to the density of soil moisture station network. The denser the soil moisture station network is, the more clearly it can represent the distribution of soil moisture in a specific area, however, the soil moisture station networks are sparse in most cases. To explore the effect of the density of soil moisture station networks on the optimal C, the variation of C calibrated by different density of soil moisture station networks’ data needs to be analyzed, for this purpose, we performed the following experiment:

Step 1: Randomly sample N soil moisture stations from all 57 stations and calibrate the optimal C using these N stations’ data.
Step 2: Repeat Step 1 M times and obtain M optimal C, let data set $S_{N}=\left\{C_{N,1},\;C_{N,1},\;\ldots ,\;C_{N,M}\right\}$, $C_{N,i}$ presents the ith optimal C corresponding the ith sampled N stations, here let M = 500.
Step 3: Upgrade N with N = N + 1 until N = 57 and get data sets $S_{1},\;S_{1},\;\ldots ,\;S_{57}$.
Step 4: The mean and inter quartile range of $S_{1},\;S_{2},\;\ldots ,\;S_{57}$ (see Figure 6) is drawn as a boxplot and the variation of data sets obtained in Step 3 are analyzed.

In this experiment, different density of station networks were simulated by changing N, the number of sampled stations. Different combinations of soil moisture stations are created by sampling several stations randomly for a given number of stations which represent different distributions of soil moisture station networks. The changes in C calibrated by different number of stations’ data can be used to analyze the variation of C. In the boxplot, the variation (from min to max) of C in each box is caused by different distributions of stations, and the range of different box represent the variation mainly caused by the density of soil moisture network. As shown in Figure 6, when the station networks are sparse, the variation within the same box is generally big, because sparse station networks can not represent the distribution of soil moisture in a specific area. With the increase of stations, in general, the variation decreases, the means of each box tend to be stable and the range of boxes tend to be smaller. Though using lots of stations help obtain an optimal C, however, the results show that not so many stations are needed for an acceptable C. Specifically, when the stations equals to 37, the means of boxes begin to be stable and the acceptable C appears. 

**Figure 7:**
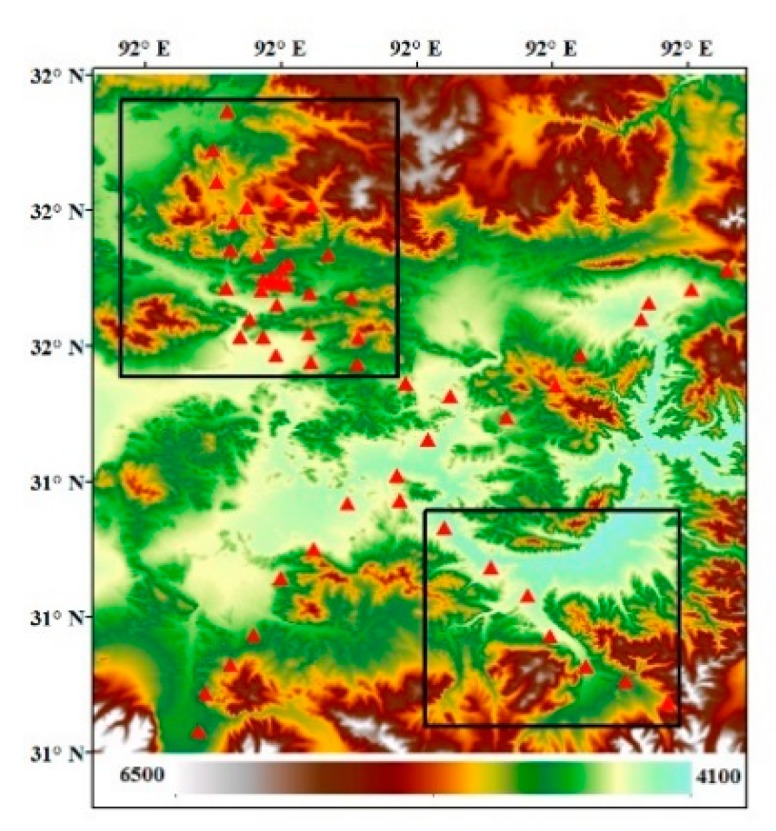
Calibration stations and validation stations, stations in black rectangles are selected as calibration stations and the others as the validation stations.

**Figure 8:**
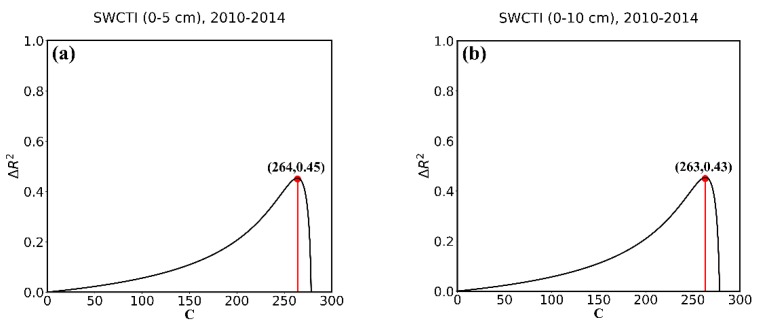
Curves of $\Delta R^{2}\;$with respect to the variable C at (**a**) 0–5 cm soil depth and (**b**) 0–10 cm soil depth.

For the purpose of the optimization of C and validation of SWCTI, we divided 57 stations into two parts, calibration stations and validation stations. To avoid the geographic autocorrelation between stations, we selected the northwest and southeast parts of the station network as the calibration stations (totally 37) and the others as the validation stations (see Figure 7). To calibrate the optimal value of C, all the data of calibration stations from 2010–2014 were used to plot the curves of $\Delta R^{2}$. Figure 8a displays the 0–5 cm in situ soil moisture data, and Figure 8b displays the 0–10 cm soil moisture data. Figure 8 show that the $\Delta R^{2}$ first increases and then decreases with the increase in C, reaching a maximum at C = 264 in Figure 8a and at C = 263 in Figure 8b. Furthermore, when C = 264, the correlation between SWCTI and the 0–5 cm soil moisture data is the highest, and when C = 263, the correlation between SWCTI and the 0–10 cm soil moisture data is the highest. Compared to F (SWCTI (C = 0)), there is a 45% and 43% increase in the coefficient of determination $R^{2}$ for the 0–5 cm and 0–10 cm soil moisture data, respectively, when the optimal C is used. 

**Figure 9:**
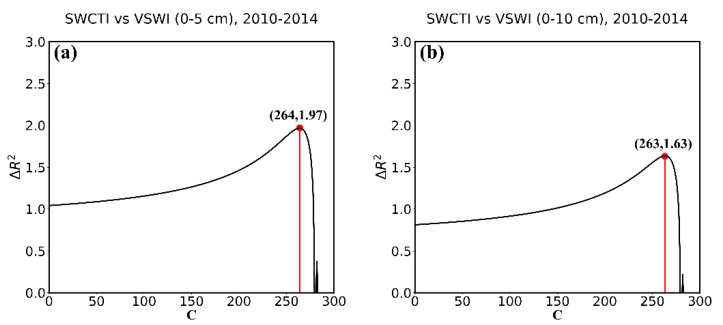
Curves of $\Delta R^{2}\;$with respect to the variable C at (**a**) 0–5 cm soil depth and (**b**) 0–10 cm soil depth.

Meanwhile, because SWCTI was developed on the basis of VSWI, we also analyzed the performance improvement of SWCTI relative to VSWI due to the use of SWCI in the numerator and C in the denominator of SWCTI. The results are shown in Figure 9; $\Delta R^{2}$ (see Equation (18)) first increases and then decreases with the increase in C and peaks at C = 264 and C = 263. In addition, the$\;\Delta R^{2}$ intercepts are 1.04 and 0.81, respectively, which means, compared to VSWI, an 104% and a 81% increase in the coefficient of determination $R^{2}$ was observed for the soil moisture at depths of 0–5 cm and 0–10 cm, respectively, caused by SWCI, because SWCI is more sensitive to NDVI. Moreover, increases of 197% and 163% at most in the coefficient of determination $R^{2}$ were observed for the soil moisture at depths of 0–5 cm and 0–10 cm, respectively, due to using SWCI and C.

According to results above, C was set to 263.5, which guaranteed the continuity of the proposed index, although this did not guarantee the optimization of the use of the C value for all cases, however, the value of C is acceptable.

(18) $$\;\Delta R^{2}=\frac{R_{C}^{2}-R_{\mathrm{VSWI}\;}^{2}}{R_{\mathrm{VSWI}}^{2}}$$

here, $R_{C}^{2}$ is the coefficient of determination $R^{2}$ of SWCTI, and $R_{\mathrm{VSWI}}^{2}$ is the coefficient of determination $R^{2}$ of VSWI. 

### 3.2. SWCTI vs. In Situ Measured Data

**Figure 10:**
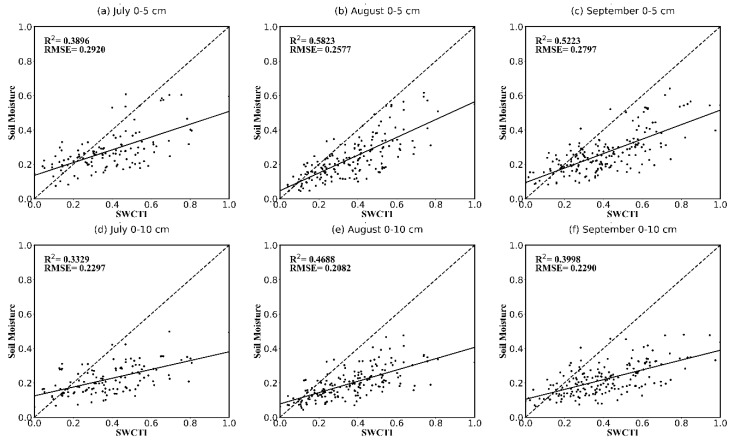
Scatterplots of SWCTI vs. in situ soil moisture for different soil depths and months, (**a**) 0–5 cm in July, (**b**) 0–5 cm in August and (**c**) 0–5 cm in September; (**d**) 0–10 cm in July, (**e**) 0–10 cm in August and (**f**) 0–10 cm in September.

To validate the performance and reliability of SWCTI, the correlations between SWCTI and the in situ soil moisture data at different soil depths (0–5 cm and 0–10 cm) in different months (July, August, and September) corresponding to validation stations, were presented using the scatterplots considering the differences of the rainfall and vegetation growth within the study area between different months (see Figure 10). In addition, SWCTI was normalized between 0 and 1 because unnormalized SWCTI values could be much less than 1. 

The results show that SWCTI has a higher correlation with the 0–5 cm soil moisture than that with at 0–10 cm. SWCTI is highly related to the 0–5 cm in situ soil moisture in August, $R^{2}=0.5823\;\left(R=0.763\right),$ and in September, $R^{2}=0.5223\;\left(R=0.723\right)$. A possible reason for the poor correlation in July is that the rainfall in Naqu City in July was, in general, significantly higher than that in August and September, causing the correlation between LST and soil moisture in July to be the lowest (see Figure 5). The average rainfall in July from 2011~2014 (there is no in situ soil moisture data in July 2010) is 140.09 mm, while the average rainfall is 97 mm in August and 80.32 mm in September of 2010~2014. Overall, SWCTI performed the best in August, followed by September and, lastly, July. A possible reason for this result is that although the vegetation began to wither in September, rainfall has a greater influence on SWCTI than vegetation growth status changes.

**Figure 11:**
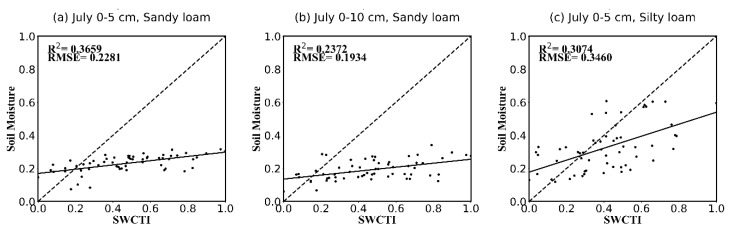

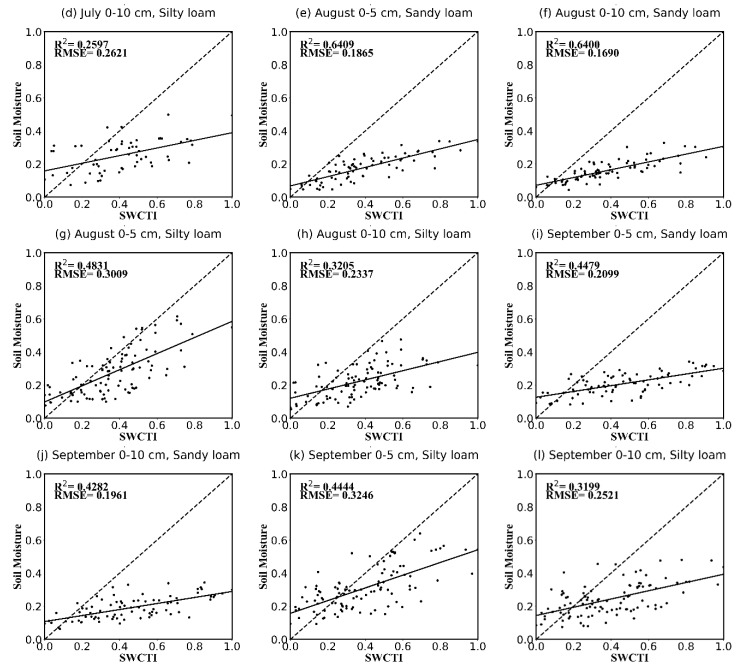
Scatterplots of SWCTI vs. in situ soil moisture for different soil depths and soil textures and different months. (**a**–**l**) are the scatterplots for the soil depths of 0–5 cm and 0–10 cm, the soil textures of sandy loam and silty loam, the months of July, August and September, respectively.

Because the spectral specifications of soil differ from soil texture, we also analyzed the correlations between SWCTI and the in situ soil moisture of different soil textures (mainly sandy loam and silty loam). The correlations are presented in Table 2 and Figure 11. The results show that SWCTI, in general, has a higher correlation with sandy loam soil moisture than silty loam soil moisture for all months; for the soil loam in August, the most significant coefficient of determination of $R^{2}=0.6409\;\left(R=0.801\right)$ was observed for the 0–5 cm soil moisture, and that of $R^{2}=0.64\;\left(R=0.8\right)$ was observed for the 0–10 cm soil moisture. 

### 3.3. SWCTI vs. Other Remote Sensing Indices

To further validate the effectiveness of SWCTI, we compared the results of SWCTI with those of several other remote sensing indices. The correlations between these remote sensing indices and soil moistures were analyzed for different soil depths, soil textures, and months. 

**Figure 12:**
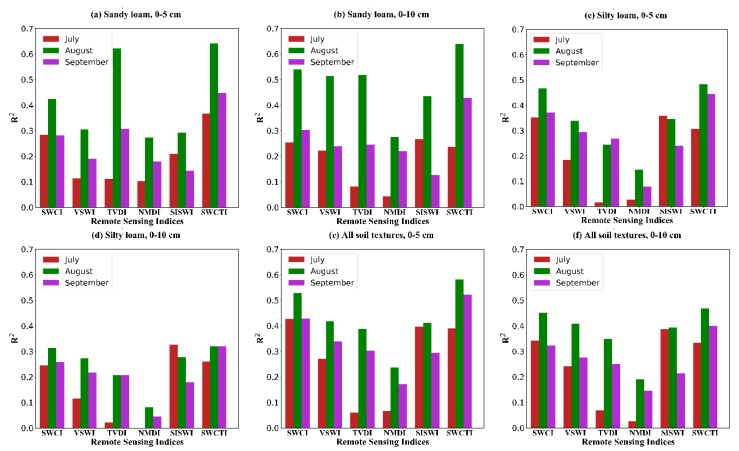
Correlation between remote sensing indices and soil moisture for different soil depths, different soil textures and different months. (**a**–**f**) show the correlation for the soil depths of 0–5 cm and 0–10 cm, the soil textures of sandy loam and silty loam and all soil textures, the months of July, August and September, respectively.

The results given in Table 3 and Figure 12 show that SWCTI has the best performance for soil moisture estimation for all cases except several cases in July, in which SIWSI or SWCI performed better and LST, as an important part of the denominator of SWCTI, performed poorly for significantly higher rainfall in July; however, the differences were small. Moreover, the correlations for August are the highest, possible reasons for this result are that the rainfall in July was significantly higher than in August and September and that the vegetation began to wither in September; therefore, the performance of remote sensing indices were affected. SWCTI is the combination of SWCI and LST, so it is affected by SWCI and LST. When the correlation between LST and soil moisture is low, the coefficient of determination of SWCTI is close to that of SWCI. However, SWCTI generally performs better than SWCI and LST.

SWCTI was developed on the basis of VSWI, because SWCI, the numerator of SWCTI, performs better than NDVI, the numerator of VSWI and the modified surface temperature, as the denominator, has a greater influence on SWCTI than that of LST; therefore, SWCI and C contributes to the improvement of SWCTI over VSWI. Moreover, VSWI is a combination of NDVI and LST, however, NDVI is a rather conservative indicator of water stress with a long time lag, because vegetation remains green after initial water stress [16,24]. It will result in overestimation of soil moisture. TVDI is based on the triangle method, determining the wet and dry edges in this index through the LST-NDVI space is a problem, to develop the TVDI using the satellite data, the image captured over a study area should contain different types of land cover and land surface condition, including both dry and wet soil, and sparse and dense vegetation coverage, however this is an ideal situation, so uncertainties will be introduced in the estimation of soil moisture using TVDI [16]. Although SWCI, SIWSI and NMDI use the water-sensitive shortwave infrared bands and can reflect the vegetation water stress, thermal bands are not included which are also sensitive to water stress [19]. The absence of the thermal bands is a limitation to their performance for soil moisture estimation. 

### 3.4. Rainfall Response

**Figure 13:**
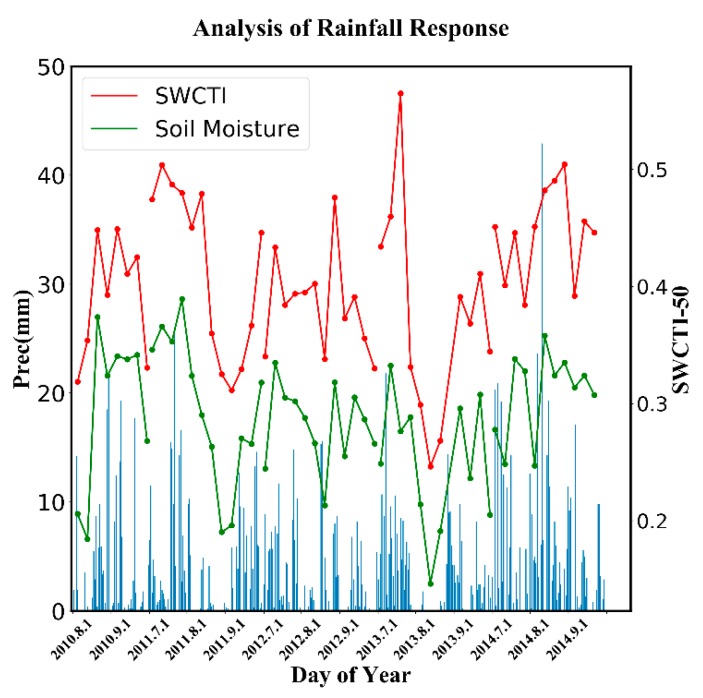
Response of SWCTI to rainfall events.

To study the sensitivity of SWCTI to rainfall events, a corresponding rainfall response analysis was carried out. Because there is only one meteorological station in the study area and a serious lack of in situ soil moisture data, we used the averages of SWCTI and soil moisture data from all soil moisture stations to present SWCTI and soil moisture over the study area. Because SWCTI was low, in order to increase the visual effect, it was scaled by a factor of 50. As shown in Figure 13, SWCTI, was generally sensitive to rainfall events, and the change in SWCTI was similar to that of the soil moisture. After rainy days, SWCTI generally showed an increasing trend (e.g., from August 2010 to September 2010, SWCTI increased). In addition, after dry days, SWCTI decreased (e.g., from July to August 2010, SWCTI decreased). SWCTI was high during the rainy seasons and low during the dry seasons.

### 3.5. Surface Soil Moisture Maps

**Figure 14:**
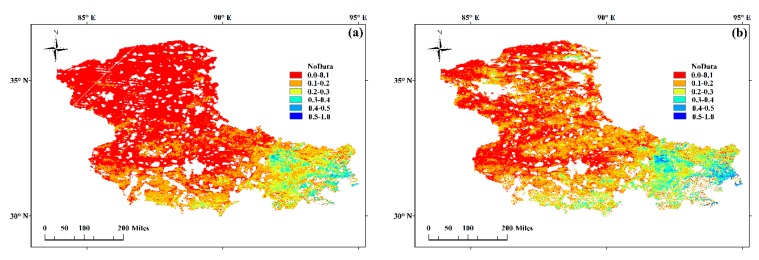
Soil moisture map at 1 km resolution over the Naqu City study area on (**a**) 5 August 2013 and (**b**) 6 September 2013.

To show the effectiveness of the proposed index, 1-km resolution surface soil moisture maps (0–5 cm depth) were generated over Naqu City by using SWCTI. Figure 14 shows the estimation results in soil moisture maps at 1 km resolution over Naqu City on 5 August (Figure 14a), 2013 and 6 September (Figure 14b), 2013. 

In Figure 14, SWCTI increases from red to blue, and the greater SWCTI is, the wetter the soil; additionally, the white regions are areas where the land cover type is not vegetation or soil moisture was not calculated because of invalid data due to clouds. As shown in Figure 14, southeastern Naqu City is wetter than other parts of Naqu, and the southern part of Naqu City is wetter than the northern part. This result is consistent with the soil moisture distribution characteristics of Naqu City. Due to the monsoon climate, rainfall is the highest in the southeast, followed by the south. Moreover, the red area in the soil moisture map for 5 August 2013 is greater than that on 6 September 2013, because dry days occurred from 29 July to 13 August, and rainy days occurred from 30 August to 14 September, which were the eight days before and after image acquisition.

## 4. Conclusions and Future Work

This paper proposed an operational and efficient surface soil moisture estimation index—SWCTI. The proposed index was calibrated and validated by in situ data and compared with other remote sensing indices. The results showed that the proposed index has a higher correlation with the surface soil moisture; the coefficient of determination between SWCTI and the in situ soil moisture reached 0.6409 (R = 0.801). Therefore, SWCTI has a great potential for use in surface soil moisture estimation.

The remote sensing indices corresponding to the sites were calculated using MODIS (500 m and 1 km resolution) data, while the in situ soil moisture data are merely representative over a very small spatial scale (point-scale); therefore, in situ data cannot represent the true condition of the soil moisture within a MODIS pixel, therefore, uncertainties were introduced during the calibration process for the proposed index. Additionally, LST has a very important influence on SWCTI as shown by the results of this study, which is related to the optimization of C factor. The poor correlation between LST and soil moisture usually results in a low correlation between SWCTI and soil moisture, therefore, any factors related to LST will have an indirect impact on SWCTI, including meteorological factors, such as clouds and rainfall. The density of soil moisture station network also influences SWCTI, because it is related to the optimization of C factor. We performed an experiment to explore how it plays, and we found that not so many stations are needed for an acceptable C. Specifically in this work, the stations equal to 37 and the means of boxes begin to be stable and the acceptable C appears. Moreover, because the denominator of SWCTI decreases, the accuracy of the LST inversion will have a greater influence on SWCTI. Meanwhile, the variable C is influenced by many other factors, such as the seasonal difference and soil textures. Our future work will focus on how to optimize C and validate SWCTI in other places in the globe.

## Figures and Tables

**Table 1:** Quality control standards of MOD09A1 and MOD11A2.

Products	Bit Number	Parameter Name	Bit Combination	Description
MOD09A1	0–1	Cloud state	00	Clear (no cloud)
	2	Cloud shadow	0	No cloud shadow
	6–7	Aerosol quantity	01	Low aerosol content
	8–9	Cirrus detected	00	No cirrus
	12	MOD35 snow/ice flag	0	No snow/ice
	13	Pixel is adjacent to cloud	0	Pixel is not adjacent to cloud
MOD11A2	0–1	Mandatory QA flags	00/01	00 = LST produced, good quality, not necessary to examine more detailed QA
				01 = LST produced, other quality, recommend examination of more detailed

Note: For a given pixel, bit number represents the position of parameter in binary-based value in the quality layer in MOD09A1 or quality layer in MOD11A2 corresponding to the given pixel. Bit combination represents the value of parameter.

**Table 2:** Correlations between SWCTIand soil moisture at different soil depths and during different months.

Soil Texture	Soil Depth	Time	RMSE	$\;R^{2}\;$
Sandy loam	0–5 cm	July	0.2281	0.3659
		August	0.1865	**0.6409**
		September	0.2099	0.4479
	0–10 cm	July	0.1934	0.2372
		August	0.1690	**0.6400**
		September	0.1961	0.4282
Silty loam	0–5 cm	July	0.3460	0.3074
		August	0.3009	**0.4831**
		September	0.3246	0.4444
	0–10 cm	July	0.2621	0.2597
		August	0.2337	**0.3205**
		September	0.2521	0.3199

**Table 3:** Correlation between remote sensing indices and soil moisture at different soil depths and during different months.

	Sandy Loam		Silty Loam		All Soil Textures	
Index	$R^{2}(5\;\mathrm{cm})$	$R^{2}(10\;\mathrm{cm})$	$R^{2}(5\;\mathrm{cm})$	R² (10 cm)	$R^{2}(5\;\mathrm{cm})$	$R^{2}(10\;\mathrm{cm})$
**July**						
NDVI	0.0876 **	0.2023	0.1778	0.1075 **	0.2588	0.2296
LST	0.2855	0.1095	0.0508 ***	0.0802 **	0.0809	0.0875
SWCI	0.2846	0.2543	0.3512	0.2463	**0.4272**	0.3416
VSWI	0.1129	0.2223	0.1843	0.1157	0.2704	0.2413
TVDI	0.1114	0.0815 **	0.0153 ***	0.0212 ***	0.0591	0.0682
NMDI	0.1026 **	0.0421 ***	0.0274 ***	0.0007 ***	0.0659	0.0262 ***
SISWI	0.2085	**0.2667**	**0.3591**	**0.3255**	0.3955	**0.3860**
**SWCTI**	**0.3659**	0.2372	0.3074	0.2597	0.3896	0.3329
**August**						
NDVI	0.2522	0.4657	0.3135	0.2558	0.3889	0.3851
LST	0.6095	0.4112	0.1970	0.1593	0.2979	0.2496
SWCI	0.4239	0.5444	0.4671	0.3141	0.5282	0.4516
VSWI	0.3041	0.5140	0.3380	0.2734	0.4178	0.4084
TVDI	0.6214	0.5180	0.2441	0.2069	0.3874	0.3486
NMDI	0.2727	0.2759	0.1457	0.0817	0.2366	0.1911
SISWI	0.2926	0.4355	0.3446	0.2778	0.4118	0.3928
**SWCTI**	**0.6409**	**0.6400**	**0.4831**	**0.3205**	**0.5823**	**0.4688**
**September**						
NDVI	0.1627	0.2146	0.2670	0.1966	0.3116	0.2539
LST	0.2271	0.1516	0.1273	0.0938	0.1544	0.1254
SWCI	0.2818	0.3023	0.3722	0.2583	0.4291	0.3234
VSWI	0.1891	0.2389	0.2944	0.2167	0.3395	0.2757
TVDI	0.3069	0.2456	0.2689	0.2065	0.3028	0.2508
NMDI	0.1786	0.2204	0.0784	0.0452 **	0.1717	0.1450
SISWI	0.1431	0.1258	0.2405	0.1801	0.2936	0.2142
**SWCTI**	**0.4479**	**0.4282**	**0.4444**	**0.3199**	**0.5223**	**0.3988**

Note: The symbol ** represents that the correlation is significant at the 0.05 level, and the symbol *** represents that the correlation is not significant at the 0.05 level, while other correlations are significant at the 0.001 level.

## Appendix A

Locations of the 57 soil moisture stations used in this paper are shown in Table A1.

**Table A1:** Locations of the 57 experimental stations.

Station ID	Longitude	Latitude	Station Name
1	92.369° E	31.0737° N	BC02
2	92.30864° E	31.10699° N	BC03
3	92.25001° E	31.12826° N	BC04
4	92.19722° E	31.17213° N	BC05
5	92.16429° E	31.23203° N	BC06
6	92.10947° E	31.27422° N	BC07
7	92.04075° E	31.33244° N	BC08
8	92.45807° E	31.71253° N	CD01
9	92.40511° E	31.68347° N	CD02
10	92.34232° E	31.66402° N	CD03
11	92.33065° E	31.63956° N	CD04
12	92.24055° E	31.58738° N	CD05
13	92.20562° E	31.54072° N	CD06
14	92.1326° E	31.49582° N	CD07
15	91.72122° E	31.94637° N	MS3475
16	91.69954° E	31.88961° N	MS3482
17	91.70566° E	31.8431° N	MS3488
18	91.74971° E	31.80555° N	MS3494
19	91.78268° E	31.75401° N	MS3501
20	91.81075° E	31.72237° N	MS3506
21	91.8424° E	31.67754° N	MS3513
22	91.79455° E	31.66171° N	MS3518
23	91.7548° E	31.63929° N	MS3523
24	91.73968° E	31.61433° N	MS3527
25	91.79339° E	31.58681° N	MS3533
26	91.84397° E	31.57667° N	MS3538
27	91.91269° E	31.57351° N	MS3545
28	91.98467° E	31.54569° N	MS3552
29	92.04956° E	31.52711° N	MS3559
30	91.97082° E	31.41003° N	MS3576
31	91.84779° E	31.30087° N	MS3593
32	91.79928° E	31.259° N	MS3603
33	91.7597° E	31.17451° N	MS3614
34	91.72559° E	31.1293° N	MS3620
35	91.68809° E	31.08875° N	MS3627
36	91.67885° E	31.03275° N	MS3633
37	92.01722° E	31.46306° N	MSNQRW
38	91.89898° E	31.36865° N	MSBJ
39	91.72979° E	31.78205° N	P1
40	91.72515° E	31.74254° N	P2
41	91.71921° E	31.68508° N	P3
42	91.91455° E	31.61232° N	P5
43	91.90449° E	31.67132° N	P7
44	91.86991° E	31.73552° N	P8
45	91.76586° E	31.73181° N	P9
46	91.84574° E	31.80793° N	P10
47	91.79579° E	31.81599° N	P11
48	91.77073° E	31.68322° N	C1
49	91.80789° E	31.6907° N	C2
50	91.77477° E	31.61436° N	C3
51	91.84091° E	31.61812° N	C4
52	91.79937° E	31.6933° N	F1
53	91.78702° E	31.7032° N	F2
54	91.80188° E	31.71593° N	F3
55	91.77358° E	31.69828° N	F4
56	91.78628° E	31.69385° N	F5
57	91.97618° E	31.37255° N	BC+

## References

[1] Legates D.R., Mahmood R., Levia D.F., DeLiberty T.L., Quiring S.M., Houser C., Nelson F.E. (2011). Soil moisture: A central and unifying theme in physical geography. Prog. Phys. Geogr..

[2] Yin Z., Lei T., Yan Q., Chen Z., Dong Y. (2013). A near-infrared reflectance sensor for soil surface moisture measurement. Comput. Electron. Agric..

[3] Zhu W., Jia S., Lv A. (2017). A time domain solution of the Modified Temperature Vegetation Dryness Index (MTVDI) for continuous soil moisture monitoring. Remote Sens. Environ..

[4] Yan F., Qin Z., Li M., Li W., International Society for Optics and Photonics (2006). Progress in soil moisture estimation from remote sensing data for for agricultural drought monitoring. Remote Sensing for Environmental Monitoring, GIS Applications, and Geology VI.

[5] Ghulam A., Qin Q., Zhan Z. (2007). Designing of the perpendicular drought index. Environ. Geol..

[6] Ghulam A., Qin Q., Teyip T., Li Z.L. (2007). Modified perpendicular drought index (MPDI): A real-time drought monitoring method. ISPRS J. Photogramm. Remote Sens..

[7] Yao Y.J., Qin Q.M., Zhao S.H., Yuan W. (2011). Retrieval of soil moisture based on MODIS shortwave infrared spectral feature. J. Infrared Millim. Wave.

[8] Ting D., Lingkui M., Wen Z. (2015). Analysis of the application of MODIS shortwave infrared water stress index in monitoring agricultural drought. J. Remote Sens..

[9] Fensholt R., Sandholt I. (2003). Derivation of a shortwave infrared water stress index from MODIS near-and shortwave infrared data in a semiarid environment. Remote Sens. Environ..

[10] Du Xiao W.S., Yi Z. (2007). Construction and Validation of a New Model for Unified Surface Water Capacity Based on MODIS Data. Geomatics Inf. Sci. Wuhan Univ..

[11] Zhang N., Hong Y., Qin Q., Liu L. (2013). VSDI: A visible and shortwave infrared drought index for monitoring soil and vegetation moisture based on optical remote sensing. Int. J. Remote Sens..

[12] Wang L., Qu J.J., Hao X. (2008). Forest fire detection using the normalized multi-band drought index (NMDI) with satellite measurements. Agric. For. Meteorol..

[13] Price J.C. (1990). Using spatial context in satellite data to infer regional scale evapotranspiration. IEEE Trans. Geosci. Remote Sens..

[14] Carlson T.N., Gillies R.R., Perry E.M. (1994). A method to make use of thermal infrared temperature and NDVI measurements to infer surface soil water content and fractional vegetation cover. Remote Sens. Rev..

[15] Moran M.S., Clarke T.R., Inoue Y., Vidal A. (1994). Estimating crop water deficit using the relation between surface-air temperature and spectral vegetation index. Remote Sens. Environ..

[16] Sandholt I., Rasmussen K., Andersen J. (2002). A simple interpretation of the surface temperature/vegetation index space for assessment of surface moisture status. Remote Sens. Environ..

[17] Nemani R., Pierce L., Running S., Goward S. (1993). Developing satellite-derived estimates of surface moisture status. J. Appl. Meteorol..

[18] Qin Q., Ghulam A., Zhu L., Wang L., Li J., Nan P. (2008). Evaluation of MODIS derived perpendicular drought index for estimation of surface dryness over northwestern China. Int. J. Remote Sens..

[19] Goetz S.J. (1997). Multi-sensor analysis of NDVI, surface temperature and biophysical variables at a mixed grassland site. Int. J. Remote Sens..

[20] Qin J., Yang K., Lu N., Chen Y., Zhao L., Han M. (2013). Spatial upscaling of in-situ soil moisture measurements based on MODIS-derived apparent thermal inertia. Remote Sens. Environ..

[21] Zhao L., Yang K., Qin J., Chen Y., Tang W., Montzka C., Wu H., Lin C., Han M., Vereecken H. (2013). Spatiotemporal analysis of soil moisture observations within a Tibetan mesoscale area and its implication to regional soil moisture measurements. J. Hydrol..

[22] Baldridge A.M., Hook S.J., Grove C.I., Rivera G. (2009). The ASTER spectral library version 2.0. Remote Sens. Environ..

[23] Zhang H., Chen H., Sun R., Yu W., Zou C., Shen S., International Society for Optics and Photonics (2009). The application of unified surface water capacity method in drought remote sensing monitoring. Remote Sensing for Agriculture, Ecosystems, and Hydrology XI.

[24] Adegoke J.O., Carleton A.M. (2002). Relations between soil moisture and satellite vegetation indices in the US Corn Belt. J. Hydrometeorol..

